# Genome-Wide Identification and Bioinformatics Analyses of Host Defense Peptides Snakin/GASA in Mangrove Plants

**DOI:** 10.3390/genes14040923

**Published:** 2023-04-16

**Authors:** Chenjing Shang, Ting Ye, Qiao Zhou, Pengyu Chen, Xiangyu Li, Wenyi Li, Si Chen, Zhangli Hu, Wei Zhang

**Affiliations:** 1 Shenzhen Key Laboratory of Marine Bioresource and Eco-Environmental Science, Guangdong Provincial Key Laboratory for Plant Epigenetics, College of Life Sciences and Oceanography, Shenzhen University, Shenzhen 518060, China; 2 College of Physics and Optoelectronic Engineering, Shenzhen University, Shenzhen 518060, China; 3 Department of Biochemistry and Chemistry, La Trobe Institute for Molecular Science, La Trobe University, Bundoora, VIC 3086, Australia

**Keywords:** host defense peptides (HDPs), mangrove plants, Snakin/GASA family members, *Avicennia marina*, *Kandelia obovata*, *Aegiceras corniculatum*, bioinformatic analyses

## Abstract

Host defense peptides (HDPs) are components of plant defensive barriers that resist microbial infection. Members of the Snakin/GASA protein family in plants have functions of regulating plant growth, defense, and bacteriostasis. Most mangrove plants grow in coastal zones. In order to survive in harsh environments, mangrove plants have evolved complex adaptations against microbes. In this study, Snakin/GASA family members were identified and analyzed in the genomes of three mangrove species. Twenty-seven, thirteen, and nine candidate Snakin/GASA family members were found in *Avicennia marina*, *Kandelia obovata*, and *Aegiceras corniculatum*, respectively. These Snakin/GASA family members were identified and categorized into three subfamilies via phylogenetic analysis. The genes coding for the Snakin/GASA family members were unevenly distributed on chromosomes. Collinearity and conservative motif analyses showed that the Snakin/GASA family members in *K. obovata* and *A. corniculatum* underwent multiple gene duplication events. Snakin/GASA family member expression in normal leaves and leaves infected with pathogenic microorganisms of the three mangrove species was verified using real-time quantitative polymerase chain reaction. The expression of *KoGASA3* and *4*, *AcGASA5* and *10*, and *AmGASA1*, *4*, *5*, *15*, *18*, and 23 increased after microbial infection. This study provides a research basis for the verification of HDPs from mangrove plants and suggests directions for the development and utilization of marine biological antimicrobial peptides.

## 1. Introduction

Plants have evolved sophisticated defense mechanisms in the natural environment to protect themselves from bacteria, fungi, viruses, and protozoa [1]. For example, in the constitutive defense, waxy cuticles and trichomes form physical barriers against the infiltration and spread of pathogenic microorganisms [2]. Alternatively, in the induced defense, by triggering a cascade of reactions that activate many defense-related genes, a variety of proteins and secondary metabolites are released to inhibit the growth of pathogenic microorganisms [3,4]. Among the defense molecules in plants, host defense peptides (HDPs) are common defense barriers that plants have evolved to resist microbial stress [5]. HDPs are part of the innate immune system inherent in almost all life forms and are found in microbes, arthropods, amphibians, mammals, and plants. HDPs significantly contribute to host defense against pathogens [6,7,8,9]. In plants, HDPs are typically peptides with masses less than 9000 Daltons; they are thermally stable and positively charged, and they have a significant proportion of hydrophobic amino acids (>30%) in linear or circular structures [5,10,11].

According to the sequence, cysteine number, and protein structure, plant HDPs can be divided into eight families: defensins, thionins, nonspecific lipid transfer proteins (LTPs), Snakins, hevein-like peptides, knottins, α-hairpinins, and cyclic peptides [12]. Snakin was first discovered in the stem of *Solanum tuberosum* and was named Snakin because of some common sequence motifs with snake venom [13,14]. Sequence analysis of potato Snakin/GASA predicted proteins showed that this family has three distinct structural domain features, and the most representative of these three subgroups are Snakin-1, Snakin-2, and Snakin-3, respectively. Snakins are typically smaller than 9000 Daltons, positively charged, and rich in cysteines [13,14]. The peptides comprise three parts: a signal peptide at the N-terminal, a variable intermediate region, and a conserved GASA domain [13,14]. The GASA (Gibberellins Stimulated in *Arabidopsis thaliana*) gene family in *A. thaliana* is consistent with the structural features of Snakin [15,16]. Increasing numbers of Snakin/GASA family members have recently been identified in various monocotyledonous and dicotyledonous plants [10,13,17]. For example, seven Snakin/GASA family members were found in *Allium cepa* L.; fourteen Snakin/GASA family members were found in *Vitis vinifera* L., and thirty-seven Snakin/GASA family members were found in *Glycine max* [18,19,20]. The present study aimed to discover and identify novel members of the Snakin/GASA family of plants in the mangroves.

Various plant hormones regulate the expression of Snakin/GASA family members. For example, gibberellins (Gas) can induce *AtGASA4*, *6*, *7*, *8*, and *13* in *A. thaliana*, *PeuGASA5*, *6*, *12*, *17*, and *19* in *Populus euphratica*, and *OsGASA1* in *Oryza sativa* [10,15,16,21]. Methyl jasmonate (MeJA) can inhibit the expression of *PeuGASA4*, *8*, *9*, and *15* in *P. euphratica* [21]. Abscisic acid (ABA) can induce the expression of *AtGASA2*, *3*, *5*, and *14* in *A. thaliana* and *PeuGASA9*, *10*, and *14* in *P. euphratica* but inhibit the expression of *AtGASA7* and *9*, *PeuGASA8*, *11*, *15*, *17*, and *18* [10,16,21]. At the same time, Snakin/GASA family members participate in various physiological processes such as plant cell division, flower induction, seed germination, and root growth. For example, overexpression of *AtGASA6* caused early flowering in *A. thaliana*. The overexpression of *GmGASA32* can promote soybean height [22,23].

Snakin/GASA family members can inhibit the growth of various bacteria and fungi at very low concentrations. *StSN1* isolated from *S. tuberosum* inhibited the growth of fungal pathogens such as *Fusarium solani*, *Fusarium culmorum*, *Bipolaris maydis*, and *Botrytis cinerea*, as well as bacterial pathogens such as *Clavibacter michiganensis* at low concentrations (EC50 < 10 μM) [14]. *PnSN1* found in *Panax notoginseng* inhibited the mycelial growth of four phytopathogenic fungi (*F. solani*, *Fusarium oxysporum*, *Fusarium verticillioides*, and *Botryosphaeria dothidea*) and the spore germination of *F. solani* [17]. *PdSN1* (*Peltophorum dubium* Snakin peptide) inhibited *Streptomyces scabies* at 1.8 μM [24]. In addition to their antifungal and antibacterial activities, the Snakin/GASA family protects plants from viral threats; for example, overexpression of *GmSN1* enhanced viral resistance in *A. thaliana* and *G. max* [18].

Mangroves form unique ecosystems in the intertidal zones of tropical and subtropical regions. The living environment of mangrove plants is more complex than that of terrestrial and aquatic plants, and thus they face more diverse pathogenic microorganisms. Therefore, there may be very efficient HDPs in mangrove plants. However, a literature search (Google Scholar; keywords: host defense peptide in mangrove plants, 28 March 2022) found few research reports concerning HDPs from mangrove plants. Therefore, we first identified Snakin/GASA family members in *K. obovata*, *A. corniculatum*, and *A. marina*. Chromosomal localization, gene expansion, gene structure, and upstream promoter cis-acting elements of candidate Snakin/GASA family members were predicted and analyzed. The chemical properties, subcellular localization, motifs, and phylogenetic relationships of the encoded proteins were also predicted and analyzed. The gene expression changes of the Snakin/GASA family were examined on the leaves of three mangrove species, which are infected by pathogenic microorganisms in their natural habitats. This study provides a resource for future research on HDPs from mangrove species and suggests directions for developing and utilizing marine biological antimicrobial peptides.

## 2. Materials and Methods

### 2.1. Gene Identification of Snakin/GASA Family in K. obovata, A. corniculatum, and A. marina

The genomic data of *K. obovata* were obtained from Genome Warehouse (GWH) with the accession code PRJCA002330/GWHACBH00000000 [25]. The *A. corniculatum* genome data were obtained from the China National GeneBank (CNGB), accession number CNA0017738 [26]. *A. marina* genomic data were obtained from NCBI (www.ncbi.nlm.nih.gov/, 3 April 2022), Genebank numbers GCA_019155195.1 (file 1, assembled to the chromosomal level but no transcripts) and PRJNA392013 (file 2, assembled to the scaffold level with transcripts [27]). The hidden Markov model (HMM) profile of the GASA domain (PF02704) from the Pfam database was used as a query, and the putative Snakin/GASA family members were identified by HMMER searching against the *K. obovata*, *A. corniculatum*, and *A. marina* genomes. The complete GASA domains of the protein sequences were examined using Batch CD-Search tools (https://www.ncbi.nlm.nih.gov/Structure/cdd/cdd.shtml, 3 April 2022) and Pfam (http://pfam.xfam.org/, 3 April 2022). Finally, conserved domains of all candidate protein sequences were verified by manually removing incomplete domain sequences.

### 2.2. Physicochemical Characterization, Chromosomal Location, and Sequence Alignments

Snakin/GASA family members’ physicochemical properties were predicted using ProtParam (https://web.expasy.org/protparam/, 3 April 2022). The chromosomal locations of Snakin/GASA family members were confirmed in the gene annotation files of *K. obovata* and *A. corniculatum* and mapped using MapChart software [28]. The coding sequences of Snakin/GASA family members were confirmed in the genome file 2 of *A. marina*. Then the chromosomal location information of Snakin/GASA family members was obtained in file 1 by blastn and visualized using MapChart software [28]. Snakin/GASA family members of the three mangrove species were aligned using Genedoc software [29] with Snakin-1, Snakin-2, and Snakin-3 sequences of *S. tuberosum*.

### 2.3. Phylogenetic, Gene Structure, Motif, and Promoter Region Analyses

A phylogenetic tree was constructed using the neighbor-joining method with 1000 bootstrap replicates via MEGAX software with Snakin/GASA sequences of *K. obovata*, *A. corniculatum*, *A. marina*, *V. vinifera*, *S. tuberosum*, *P. euphratica*, *A. thaliana*, *Malus domestica*, and *Populus trichocarpa*. EVOLVIEW (https://www.evolview.com, 3 April 2022) was used to visualize the evolutionary tree. Gene structure was analyzed using GSDS tools (http://gsds.cbi.pku.edu.cn, 5 April 2022). Motifs in sequences were analyzed using the MEME online tool (http://meme-suite.org/, 5 April 2022). Prediction of cis-acting elements was performed on the upstream sequence of each gene (1.5 KB) using the PlantCARE online tool (http://sphinx.rug.ac.be:8080/PlantCARE/, 6 April 2022), and the final images were created using TBtools software [30].

### 2.4. Subcellular Localization, Protein Structure and Gene Duplication

Subcellular localization analysis of GASA was performed using the online sites WoLF PSORT (https://www.genscript.com/wolf-psort.html, 7 April 2022) and Plant-PLoc (http://www.csbio.sjtu.edu.cn/cgi-bin/PlantPLoc.cgi, 7 April 2022). Protein 3D structure prediction was performed with SWISS-MODEL (https://swissmodel.expasy.org/, 7 April 2022) and illustrated with Cherima1.14. The occurrence and duplication events of the Snakin/GASA family members in the three mangrove species were analyzed and visualized by MCScanX (University of Georgia, Athens, GA, USA). Non-synonymous (ka) and synonymous (ks) substitution rates of each duplicated WRKY gene were calculated using KaKs_Calculator 2.0.

### 2.5. Sample Collection

Plant samples obtained in the field can provide a true and visual picture of plant populations’ health status, the causes of disease, and plant response characteristics. In November 2021, infected and normal leaves of three mangrove species (Figure S1) were collected in Baguang Yinye Wetland Park (22°38′ N, 114°24′ E), Shenzhen. After washing the leaf surface with sterile water, the collected samples were photographed (Figure S1), wrapped in tinfoil, and immediately frozen in liquid nitrogen and stored at −80 °C.

### 2.6. RNA Extraction, cDNA Synthesis, and qRT-PCR Analysis

RNA was extracted using an RNA Pure Plant Plus Kit (Tiangen Biotechnology, Beijing, China). According to the manufacturer’s instructions, the extracted RNA was reverse transcribed into cDNA using the Hifair III 1st Strand cDNA Synthesis SuperMix for qPCR (YEASEN, Shanghai, China). NanoDrop nucleic acid concentration tester (Thermo Fisher Scientific, MA, USA) was used to determine the concentration and purity of RNA. Only samples with OD 260/OD 280 ratios between 1.8 and 2.0 could proceed to the next step. RNA samples were randomly selected and electrophoresed on 0.8% agarose gels to check the integrity of the RNA. The loading volume was 1 μg total RNA, and the voltage was 6 V/cm. The specific primers for Snakin/GASA family members from three mangrove species were designed using Premier 6.0. The specificity of primers was determined by 0.8% agarose gel electrophoresis. Real-time PCR was run with the qTower3 instrument (Analytik Jena AG, Jena, Germany) to detect the chemical SYBR Green. The established reaction system was as follows: 5 μL 2 × SYBR Green Pro Taq HS Premix (Accurate Biotechnology, Hunan, China), 0.5 μL of each forward and reverse primer (10 μM), 1 μL of diluted cDNA template, and RNase-free ddH_2_O were added until the total volume was 10 μL. The reaction procedure was as follows: 95 °C for 15 min, 40 cycles of 10 s at 95 °C, 15 s at 59 °C, and 20 s at 72 °C. The melting curve analysis was performed immediately after the PCR reaction, i.e., fluorescence intensity was measured for each degree increase from 60 °C to 95 °C. The relative template abundance in each PCR expansion mixture was calculated by the 2^−ΔΔCT^ method. Three biological replicates were used for gene expression analysis, and the expression of the control samples was set to 1 for normalization. Data manipulation and structural visualization were performed with GraphPad Prism 6, and an LSD test was used to calculate the *p*-value. All primers for internal reference genes and Snakin/GASA family members in the three mangrove species are shown in Tables S1 and S2. Internal reference genes refer to Dr. Peng Yalan’s research [31,32,33].

### 2.7. Identification of Pathogenic Microorganism

The infected part was cut out, and the Ezuo Column Fungal Genomic DNA Extraction Kit DNA (Sangon Biotech, Shanghai, China) instructions were followed to obtain. PCR amplification was performed using the fungus ITS universal primer pair (ITS1F: CTTGGTCATTTAGAGGAAGTAA; ITS4R: TCCTCCGCTTATTGATATGC) that amplifies the ITS1-5.8S-ITS2 region, and the bacteria 16S universal primer pair (27F: AGAGTTTGATCMTGGCTCAG; 1492R: TACGGYTACCTTGTTACGACTT) that amplifies the V1-V5 variable regions. The nucleic acid was recovered from an agarose gel and sent for sequencing. The sequencing results were compared to the NCBI database to identify the pathogenic microbes.

### 2.8. Statistical Analysis

GraphPad Prism 7.0 (GraphPad, San Diego, CA, USA) was selected for statistical analysis. Statistical significance was determined via a one-way analysis of variance. All data are presented as mean ± SE and *p*-value < 0.05 was considered statistically significant.

## 3. Results

### 3.1. Identification of Snakin/GASA Genes in Three Mangrove Plants and Chromosomal Location

**Figure 1:**
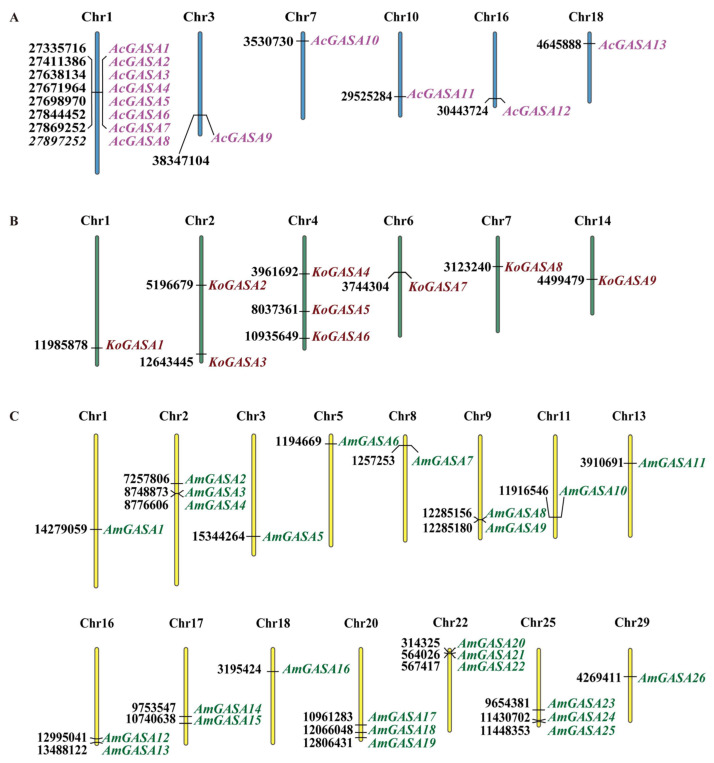
Chromosomal locations of Snakin/GASA family members in three mangrove species. (**A**) Chromosomal locations of Snakin/GASA family members in *A. corniculatum*. (**B**) Chromosomal locations of Snakin/GASA family members in *K. obovata*. (**C**) Chromosomal locations of Snakin/GASA family members in *A. marina*.

Chromosome-scale assemblies of the genomes of *K. obovata* and *A. corniculatum* were reported in 2020 [25] and 2021 [26]. A reference-grade genome of *A. marina* was published in 2021 [27]. We first used the conserved GASA domain (PF02704) to search for Snakin/GASA family members in the three mangrove species using Hmmersearch. Thirteen candidate Snakin/GASA family members were found in *A. corniculatum*; nine candidate Snakin/GASA family members were found in *K. obovate*; and twenty-seven candidate Snakin/GASA family members were found in *A. marina*. The Snakin/GASA family members of the three mangrove species were named according to their locations on the chromosomes (Figure 1). As shown in Figure 1, the *A. corniculatum* genome contained 13 Snakin/GASA family members spread across six chromosomes. Eight Snakin/GASA family members of *A. corniculatum* (*AcGASA1*–*AcGASA8*) were located on chromosome 1. Nine Snakin/GASA family members were spread across six chromosomes in the *K. obovata* genome. On chromosomes 4 and 2, there were three and two Snakin/GASA family members, respectively. *AmGASA27* was not placed on a scaffold and could not be located on a chromosome. The remaining 26 Snakin/GASA family members were distributed on 15 chromosomes in the *A. marina* genome. Furthermore, three Snakin/GASA family members were respectively identified on *A. marina*’s chromosomes 2, 20, 22, and 25.

### 3.2. Physicochemical Properties Prediction

As shown in Table 1, after ProtParam prediction, among the Snakin/GASA family members in *A. marina*, AmGASA22 had 88 amino acids and was the smallest. AmGASA19 had 307 amino acids. Among the Snakin/GASA family members of *K. obovata*, KoGASA9 was the largest with 186 amino acids. There were 421 amino acids in AcGASA6 of *A. corniculatum*, the Snakin/GASA family member with the most significant number of amino acids among the three mangrove species. AcGASA7 and AcGASA8 had 346 and 387 amino acids, respectively. KoGASA1, 4, 5, and 8 in *K. obovata*, AcGASA5, 9, 10, 12, and 13 in *A. corniculatum*, and AmGASA2, 5, 11, 16, 17, 21, 22, and 23 in *A. marina* had fewer than 100 amino acids. 

Table 1 displays the predicted properties of Snakin/GASA family members in three different mangrove species. The majority of these proteins were classified as basic proteins with a theoretical isoelectric point (pI) value above 8. The proteins AmGASA4 and AmGASA19 had the highest and lowest pI values, with 9.83 and 5.39, respectively.

### 3.3. Subcellular Localization Prediction

This study predicted the subcellular localization of Snakin/GASA family members in three mangrove species. For *A. corniculatum*, AcGASA2, 5, 10, and 12 were predicted to be localized in the extracellular matrix, AcGASA6 in the nucleus, and AcGASA3, 4, 8, and 9 in the chloroplast (Table S3). AcGASA1, 11, and 13 were predicting localized in the chloroplast and extracellular matrix. In *K. obvoata*, the predicted localization of KoGASA2, 4, 5, 6, 7, and 8 was in the extracellular matrix. KoGASA1 was distributed in the extracellular and Golgi apparatus. KoGASA9 was localized in the mitochondrion and chloroplast. In *A. marina*, AmGASA3 and 12 localized in the cytoplasm. The predicted localization of AmGASA1, 4–6, 8–11, 14–18, 20, 21, 23, and 27 was in the extracellular matrix. 

### 3.4. Phylogeny, and Protein Sequence Analysis of Snakin/GASA Family Members in Three Mangrove Species

**Figure 2:**
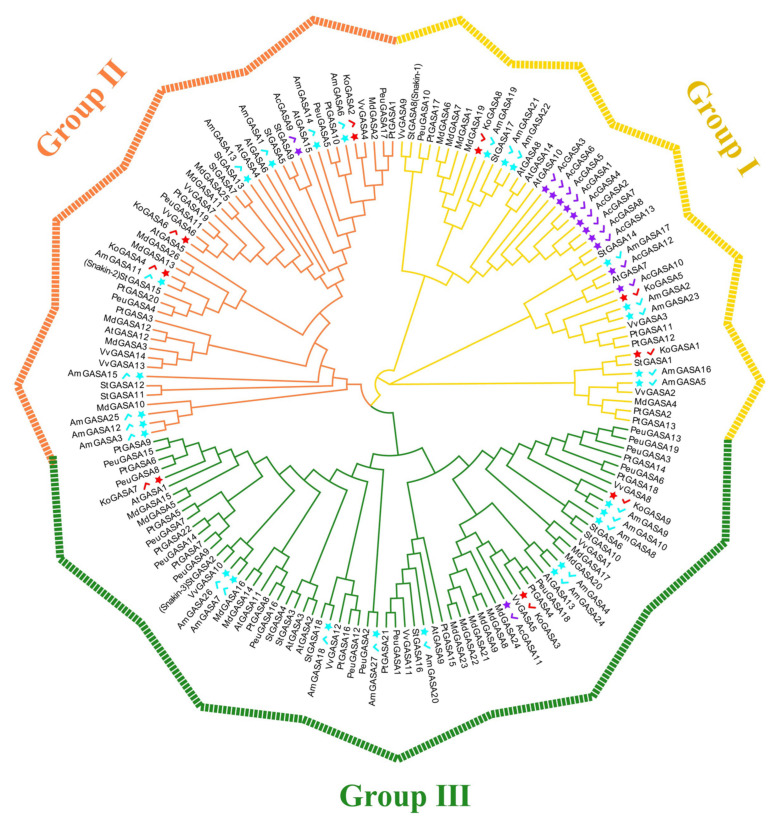
Phylogenetic tree of Snakin/GASA family members of *A. corniculatum*, *K. obovata*, *A. marina*, *V. vinifera*, *S. tuberosum*, *P. euphratica*, *A. thaliana*, *M. domestica*, and *P. trichocarpa*. Members of the *A. corniculatum* family are denoted by purple stars and tick marks, while red and blue indicate the *K. obovata* and *A. marina* families, respectively.

To illustrate the evolutionary relationships of the Snakin/GASA family members of the three mangrove species, a phylogenetic tree of conserved GASA domains was constructed (Figure 2). The GASA domains of all species were divided into three groups. Group III had the most members. There were 11 Snakin/GASA family members of *A. corniculatum* belonging to Group I. There were nine Snakin/GASA family members of *K. obovata* distributed among the three groups. There were 8, 6, and 13 Snakin/GASA family members of *A. marina* in groups I, II, and III, respectively. The three mangrove plants were more phylogenetically related to *V. vinifera* L. and *P. trichocarpa*.

**Figure 3:**
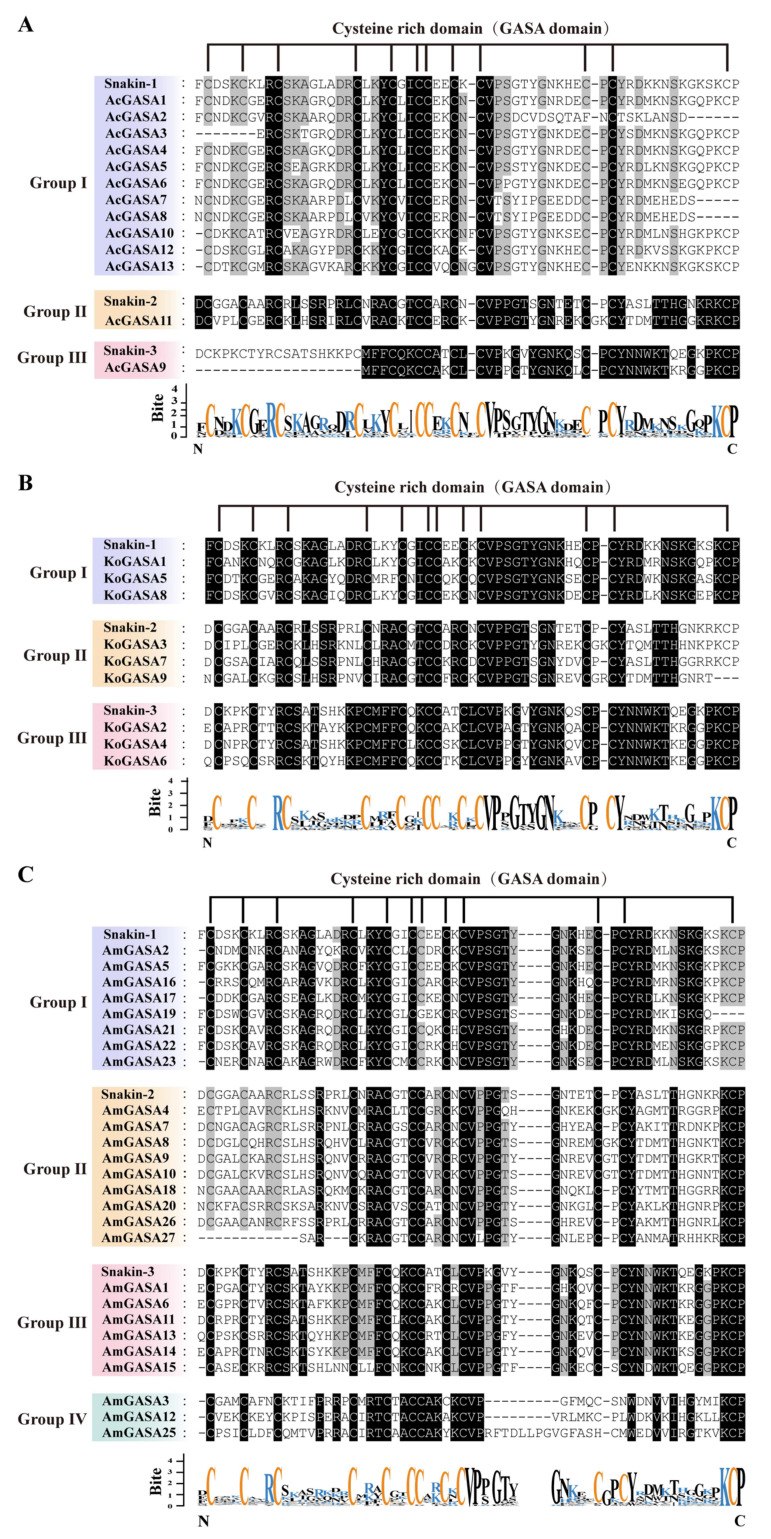
The GASA domains of Snakin/GASA family members from three mangrove species (*A. corniculatum* (**A**), *K. obovate* (**B**), and *A. marina* (**C**)) were compared with those of potato Snakins.

The Snakin-1, Snakin-2, and Snakin-3 of potato were aligned with the Snakin/GASA family members of the three mangrove species. The Snakin/GASA family members of *A. corniculatum* and *K. obovata* were divided into three groups (Figure 3A,B). Snakin/GASA family members of *A. marina* were divided into four groups. Sequence analysis of the predicted proteins of *A. marina* species showed that the family has four distinct structural domain features, while the fourth family members are missing some of the conserved domains. AmGASA3, 12, and 15 were dissimilar from the three Snakins of potato (Figure 3C). KoGASA4 and AmGASA11 were adjacent to StGASA15 (Snakin-3); AmGASA7 and AmGASA26 were adjacent to StGASA2 (Snakin-2).

### 3.5. Collinearity Analysis among Snakin/GASA Family Members

**Figure 4:**
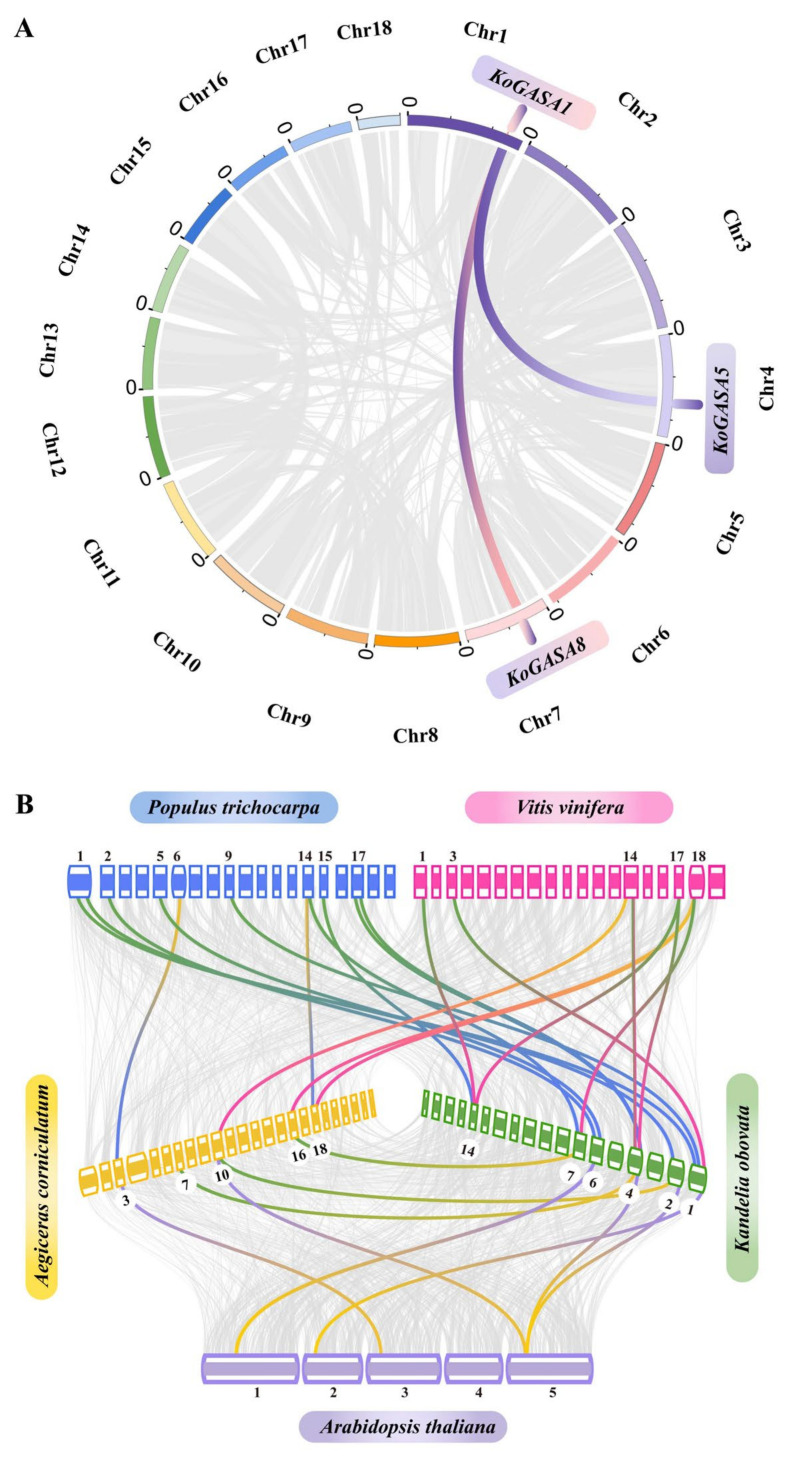
Analysis of evolutionary relationships among Snakin/GASA family members. (**A**) Fragment duplication events within the genome of *K. obovata*. (**B**) Synteny analysis of Snakin/GASA family members between *K. obovata*, *A. corniculatum*, *V. vinifera*, *P. trichocarpa*, and *A. thaliana*.

There are two versions of the *A. marina* genome. The annotation of the genome assembled to the scaffold level was relatively complete, while the annotation of the genome assembled to the chromosome level was lacking. Analyzing the intergenic collinearity of Snakin/GASA family members in the *A. marina* genome is temporarily impossible. To illustrate the family expansion pattern of Snakin/GASA family members in the three mangrove species, we analyzed gene duplication events in their genomes by McsanX. In the *K. obovata* genome, two repetitive events occurred in the evolution of Snakin/GASA family members (Figure 4A). No gene tandem duplication events were found in *K. obovata*. In *A. corniculatum*, nonsynonymous substitution rate (Ka) and synonymous substitution rate (Ks) and their ratio were estimated for Snakin/GASA family members. The gene duplication event of Snakin/GASA family members occurred on chromosome 1 (Table 2). The negative Ka/Ks values of two duplicated gene pairs (*AcGASA4* and *AcGASA3*, *AcGASA5* and *AcGASA9*, Ka/Ks values < 1) suggested negative or purifying selection pressure during evolution. T = Ks/2r, where r is the expected clock sample rate of synonymous substitution in dicotyledons, and r = 1.5 × 10^8^ substitutions/synonymous sites/year is the formula used to determine divergence time. The range of divergence times of two gene pairs calculated using Ks values was 32.28 to 33.13 million years ago (MYA) (Table 2). *AcGASA5* and *AcGASA6* had signatures of neutral evolution, as suggested by Ka/Ks values equal to 1, while the Ka/Ks values of other gene pairs were greater than one, suggesting positive selection.

Figure 4B and Table 3 display the collinearity analysis of Snakin/GASA family members among different species. Four collinear gene pairs were identified between *K. obovata* and *A. thaliana*. Between *A. corniculatum* and *A. thaliana*, two collinear gene pairs of Snakin/GASA family members were found. Between *K. obovata* and *P. trichocarpa* and *K. obovata* and *V. vinifera*, there were nine and seven collinear gene pairs of Snakin/GASA family members, respectively. Two and three collinear gene pairs of Snakin/GASA family members existed between *A. corniculatum* and *V. vinifera*, respectively. Finally, *K. obovata* and *V. vinifera* shared three gene pairs with Snakin/GASA family traits. 

### 3.6. Gene Structure and Motif Identification among Snakin/GASA Family Members

**Figure 5:**
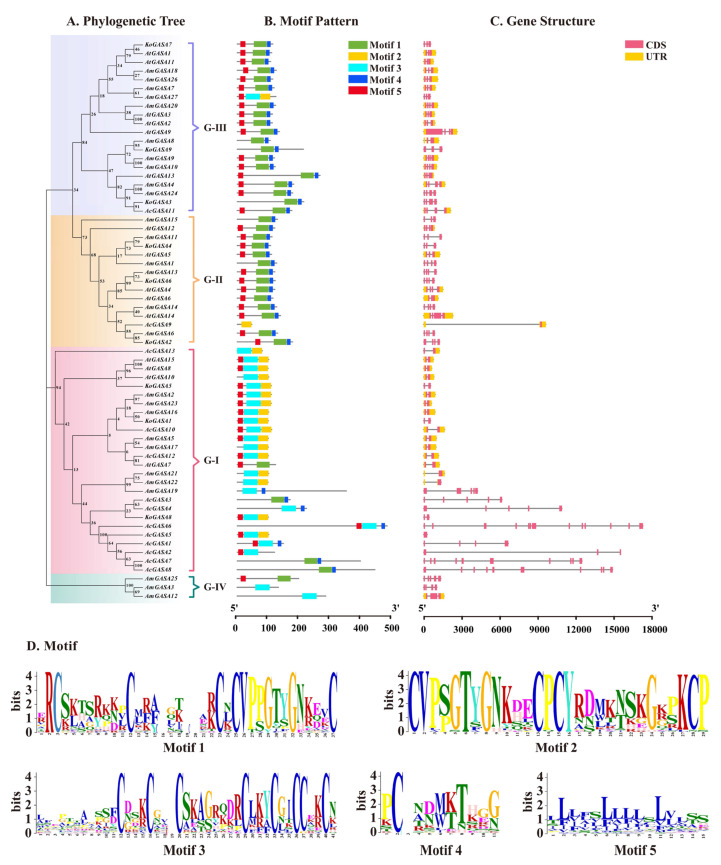
The gene structure and motif analysis of Snakin/GASA family members of three mangrove species. (**A**) Unrooted phylogenetic tree constructed based on Snakin/GASA family members of the three mangrove species and *A. thaliana*. (**B**) Conserved motif distribution of Snakin/GASA family members in the three mangrove species. (**C**) Exon–intron composition analysis. The red boxes and black lines denote exon and intron positions, respectively. (**D**) Details of conserved motifs.

As shown in Figure 5, Snakin/GASA family members in G-I of *K. obovata* consisted of two exons and introns. Other Snakin/GASA family members of *K. obovata* had multiple exons and introns. Eight Snakin/GASA family members on chromosome 1 of *A. corniculatum* did not have a UTR and had many introns and exons. *AcGASA6* of *A. corniculatum* had the largest number of introns and exons, with 12 exons and 11 introns. *AmGASA21* and *AmGASA22* of *A. marina* had only one intron and one exon, while *AmGASA19* had the largest number of exons and introns, with five exons and four introns.

The GASA domain of most Snakin/GASA family members in G-I consisted of motif 3 and motif 2 or motif 3 and motif 4. The GASA domains of *AcGASA3*, *AcGASA7*, and *AcGASA8* consisted of motif 1 and motif 4. The GASA domain of most Snakin/GASA family members in G-II and G-III consisted of motif 1 and motif 4, except for *AmGASA27* and *AcGASA9*. The GASA domains of *AcGASA9*, *AmGASA3*, *AmGASA12*, and *AmGASA25* had deletions compared to the conserved GASA domain.

### 3.7. Gene upstream Element Analysis 

**Figure 6:**
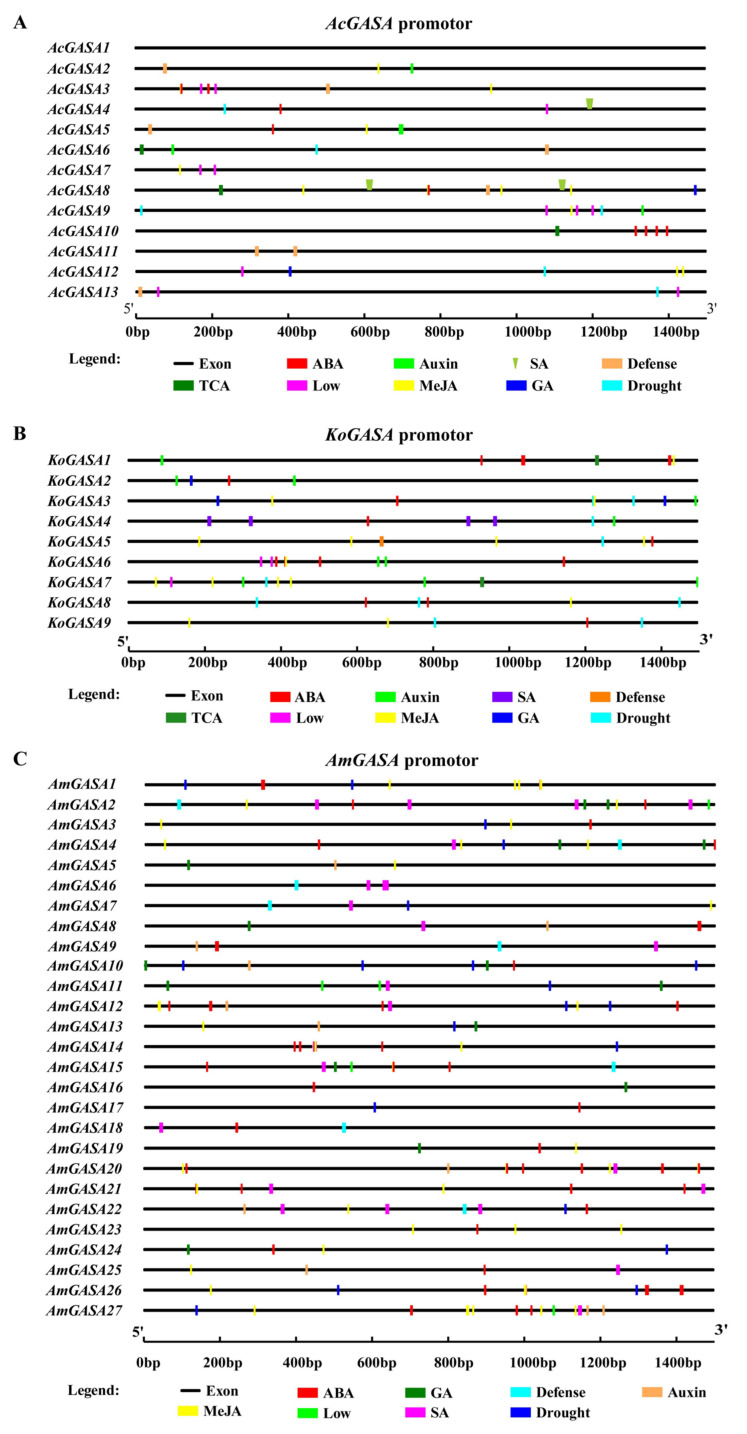
Upstream regulatory elements analysis of Snakin/GASA family members in *A. corniculatum* (**A**), *K. obovate* (**B**), and *A. marina* (**C**).

*AcGASA2*, *3*, *5*, *7*, *8*, *9*, and *12* had methyl jasmonate (MeJA) elements upstream. *AcGASA4*, *8* had SA elements upstream. *AcGASA3*, *4*, *5*, *8*, and *10* had abscisic acid (ABA) elements upstream, and there were four ABA regulatory elements upstream of *AcGASA10*. *AcGASA2*, *3*, *5*, *6*, *8*, *11*, and *13* had regulatory elements that could respond to defense (Figure 6A). MeJa regulated all Snakin/GASA family members of *K. obovata* except for *KoGASA2* and *KoGASA4*. The upstream regions of Snakin/GASA family members in *K. obovata* except for *KoGASA1*, *KoGASA2*, and *KoGASA6* had regulatory elements that could respond to drought stress. There were four salicylic acid (SA) regulatory elements upstream of *KoGASA4*. All Snakin/GASA family members of *K. obovata* were regulated by ABA except for *KoGASA8* (Figure 6B). ABA regulated all Snakin/GASA family members of *A. marina* except for *AmGASA5*-*7*, *11*, and *13*. *AmGASA1*-*7*, *10*, *12*, *13*, *15*, *18*, *21*, *23*, and *25* had MeJA and defense regulatory elements upstream (Figure 6C). 

### 3.8. Protein Structure Prediction

**Figure 7:**
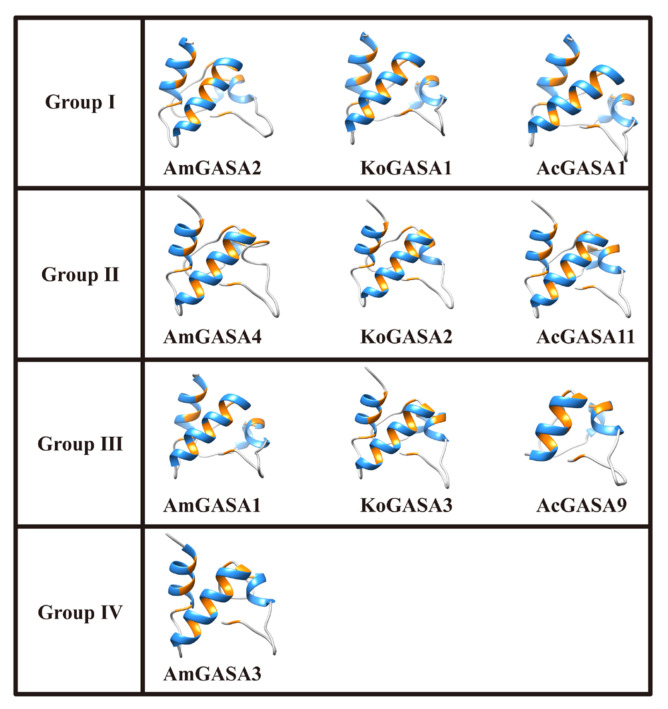
Three-dimensional protein structure prediction of partial Snakin/GASA family members of three mangrove species.

One representative of each Snakin/GASA family member in each mangrove species was selected for three-dimensional (3D) protein structure prediction. The predicted structures of these Snakin/GASA family members were similar, with all of them having random coils, extended strands, and two long α-helices (Figure 7).

### 3.9. Responses to Pathogenic Microbial Threats

To investigate the role of the GASA family in response to environmental stress, infected leaves were collected from the Dapeng mangrove forest (22°38′ N, 114°24′ E) in Shenzhen, China (Figure S1). 

Microbiological testing of black spots on collected environmental leaf samples revealed that fungal infections caused black spots on the leaves, while no bacterial infections were detected (Table S4). It was found that *Tropicoporus texanus* caused marginal and vein scorching in *A. corniculatum*. *K*. *obovata* became ulcerated as a result of *Jattaea* spp. infection. It should be *Berkeleyomyces basicola* that caused the infection of *A. marina* (Supplementary Tables S4 and S5). The expression levels of Snakin/GASA family members in the infected leaves were measured to explore their role in responding to fungal infections. 

**Figure 8:**
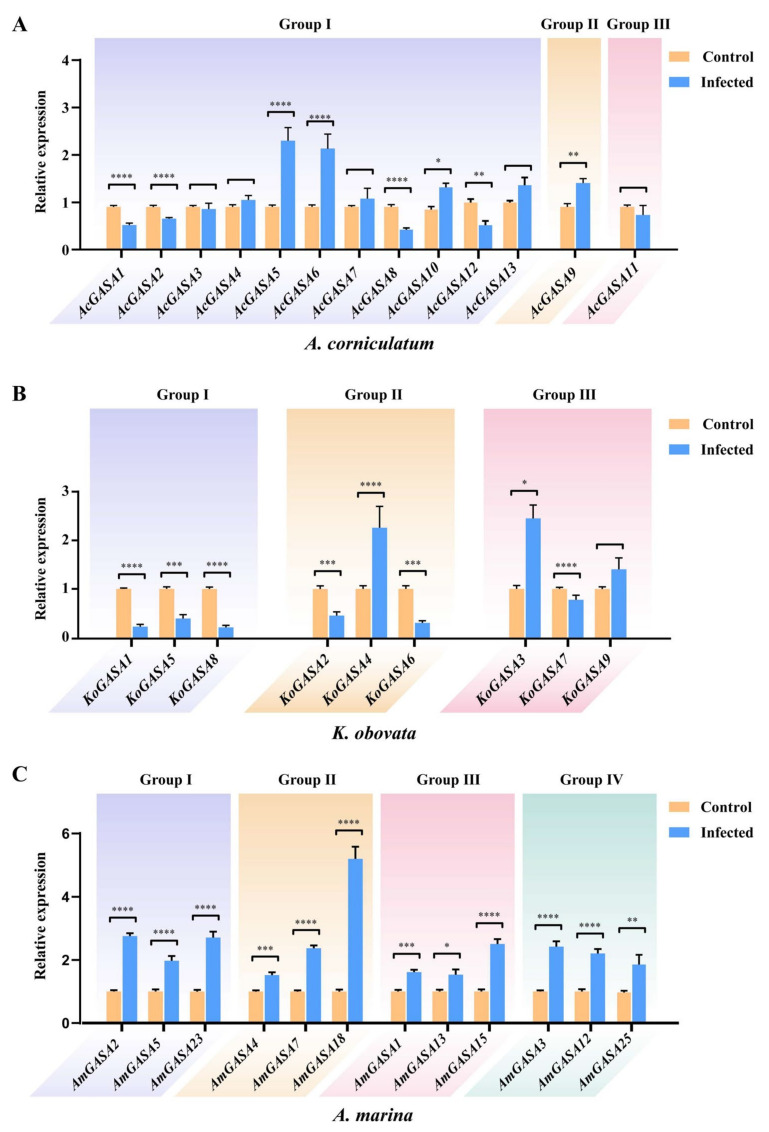
Effects of pathogenic microbial infestation on the expression of Snakin/GASA family members in three mangrove species. Differential gene expression of Snakin/GASA family members in both normal and pathogen-infected leaves of *A. corniculate* (**A**), *K. obovata* (**B**), and *A. marina* (**C**). * *p*-value < 0.05, ** *p*-value < 0.01, *** *p*-value < 0.001, **** *p*-value < 0.0001.

After collecting infected and normal leaves of three mangrove plants in the field, qRT-PCR analysis was performed to examine the expression of Snakin/GASA family members (Figure 8A–C). In the infected leaves of *A. marina*, the expression of all selected Snakin/GASA family members was significantly up-regulated, with *AmGASA18* showing the most significant difference. Compared with the normal leaves, the expression of *AmGASA18* in the leaves infected by pathogenic microbial was increased by more than fivefold. In *A. corniculatum*, *AcGASA1*, *2*, *8*, and *12* were significantly down-regulated in the infected leaves. Compared with normal leaves, the expression levels of *AcGASA5*, *AcGASA6*, *AcGASA9*, and *AcGASA10* in infected leaves were significantly increased by 2.820, 2.123, 1.563, and 1.819-fold, respectively. *AcGASA5* showed the most intense response to infection. In *K. obovata*, *KoGASA1*, *2*, *5*, *6*, *7*, and *8* were significantly down-regulated after being infected by pathogenic microorganisms. *KoGASA3* and *KoGASA4* were significantly up-regulated by 2.453 and 2.261-fold, respectively, in infected leaves. 

## 4. Discussion

Most members of the Snakin/GASA family are small peptides with multiple functions. They are regulated by various hormones involved in plant development, stress response, and antibacterial activities [10,14]. The biological processes, physicochemical properties, and gene structures of Snakin/GASA family members in various plants (including *G. max*, *V. vinifera*, and *Populus* spp.) have been reported [18,19,21]. 

Peptide lengths vary greatly among Snakin/GASA family members in the same plant species. For instance, the smallest Snakin/GASA protein in *A. thaliana* has 87 amino acids, while the largest has 275 amino acids [34]. The smallest Snakin/GASA family member in wheat has 261 amino acids, while the largest has 1099 amino acids [35]. The peptide lengths of the Snakin/GASA family members of the three mangroves varied significantly. HDPs are typically proteins with less than 100 amino acids [36]. However, many proteins with more than 100 amino acids have good antibacterial effects and may have other biological functions [37]. KoGASA1, 4, 5, and 8 in *K. obovata*, AcGASA5, 9, 10, 12, and 13 in *A. corniculatum*, and AmGASA2, 5, 11, 16, 17, 21, 22, and 23 in *A. marina* had fewer than 100 amino acids. Their amino acid numbers met the criteria for HDPs, but their antibacterial functions remain elusive.

In cotton and potato, longer chromosomes did not necessarily contain more Snakin/GASA family members. This suggests that the number of Snakin/GASA family members on each chromosome of the three mangrove species was unrelated to chromosome length. Additionally, the pI values (from 4.11 to 10.14) also vary widely among different plant species and individual members [10]. The apple MdGASA23 is currently the known Snakin/GASA family member with the lowest pI value (pI value = 4.11) [38]. The non-alkaline Snakin/GASA family members in the three mangrove species may have different functions compared to the basic Snakin/GASA family members. Typically, HDPs usually contain more cationic amino acid residues and are basic [36,37]. Therefore, electrically neutral AmGASA19 and AcGASA6 may not have normal HDP functions.

The GASA domain of Snakins is typically situated at the C-terminal and consists of approximately 60 residues, including 12 conserved cysteines [10,39]. The GASA domains of KoGASA9, AcGASA7, 8, and AmGASA19 are not located at their C-terminal. Based on their sequence features, they may not be considered part of the Snakin/GASA family. Subcellular localization provides insights into the functions of proteins [40,41,42]. Existing studies have shown that Snakin/GASA family members are distributed in various locations within plants, including nuclei, cell walls, cytoplasm, and extracellular spaces [10,17,21,43]. For example, AtGASA5 of *A. thaliana* is present in the cell wall and extracellular matrix [10,44], and HbGASA5 and HbGASA9 proteins of *Hevea brasiliensis* are present in the nucleus and the cytoplasm [45]. The transition between the cell periphery and the nucleus may be important concerning their antimicrobial function [10]. Based on the predictions, several members of the Snakin/GASA family in the three mangrove species, including AmGASA1, 4–6, 8–11, 14–18, 20, 21, 23, 27, KoGASA2, 4–8, AcGASA2, 5, 10, and 12, may have antimicrobial function and are predicted to be located in the extracellular space. KoGASA9 and AcGASA6-8 did not have signal peptides, which may be the reason why they were predicted to be localized in the intracellular space. The subcellular localization of proteins can be influenced by various factors, including post-translational modifications, electrostatic interactions, and covalent bonds with membrane lipids [10,13]. As a result, the predictions of the two methods were somewhat inconsistent. At present, it is not possible to conclusively determine whether Snakin/GASA family members have antibacterial function only based on subcellular localization.

The adjacent Snakin/GASA family members in the evolutionary tree had similar sequences and thus may have similar functions. Although many plant Snakin/GASA family members have been identified, few have been functionally validated [16,19,44,46]. The tandem and segmental duplication of genes play important roles in functional regulation, domestication, evolution, and response to biotic and abiotic stresses [42,47,48]. Segmentally duplicated genes also show similar functions and stable expression [18,49]. The motif and exon–intron analyses and Ka/Ks analysis showed that Snakin/GASA family members of *A. corniculatum* may have evolved leading to variation in motifs and introns in some groups. Some members of the Snakin/GASA family can be strongly induced to respond to temperature variation [10]. The Oligocene (33 million to 23 million years ago) was an important turning point in the rapid transformation of the Earth’s climate from “greenhouse” to “ice chamber” [50], which may be the reason for negative or purifying selection pressure during the evolution of GASA/Snakin family members in *A. corniculatum*. *AtGASA4*, *6*, and *14* in *A. thaliana* are critical in promoting plant development [16,34,51]. Inhibition of both *AtGASA4* and *6* has been shown to cause delayed flowering [16]. *KoGASA6* and *AmGASA13* are adjacent to *AtGASA4* and *6* (Figure 2), and thus they may have the same function in regulating flower development. *AtGASA4* and *AtGASA14* can interact with the cell membrane-localized receptor-like kinase protein VH1/BRL2, which participates in leaf vein development [34,51]. *KoGASA4* and *AmGASA11* are adjacent to *AtGASA5*, which are negative regulators of GA-induced flowering and stem growth [44]. *KoGASA6* is adjacent to *VvGASA7*, which regulates seed development, and they are collinear genes (Figure 2 and Table 3). Therefore, *KoGASA6* may regulate seed development [19]. *VvGASA5* can regulate ovule abortion. *AcGASA11* and *VvGASA5* are collinear genes with the same function [19]. *VvGASA2* is thought to play a role in seed development [19]. Collinear relationships were observed between *VvGASA2* and *KoGASA1*, *KoGASA5* and *AcGASA10*, and *KoGASA8* and *AcGASA12*. *KoGASA1* and *KoGASA5*, *KoGASA1* and *KoGASA8*, are intra-genome collinear gene pairs that belonged to the same group in the evolutionary tree; their amino acid sequences consisted of motifs 2, 3, and 5, and their patterns of response to pathogenic microorganisms were consistent. This suggests that their functions may be similar. Therefore, *KoGASA1*, *5*, *8*, and *AcGASA10* and *12* may regulate seed development. Snakin-1, Snakin-2, and Snakin-3 had good antibacterial effects. *KoGASA4* and *AmGASA11* were adjacent to *StGASA15* (Snakin-3); *AmGASA7* and *AmGASA26* were adjacent to *StGASA2* (Snakin-2). Therefore, *KoGASA4*, *AmGASA7*, *11*, and *26* may have the same function as Snakin-2 and Snakin-3.

Snakin/GASA family members are regulated by gibberellin (GA), abscisic acid (ABA), and other plant hormones [10,19]. The predictions via the Plant CARE website suggested that the promoters of the three mangrove GASA family members had significant differences in the number of cis-acting elements in response to abscisic acid, gibberellin, methyl jasmonate, low temperature, drought, and pathogenic microorganisms. MeJA, as a damage-related plant hormone and signaling molecule, can stimulate the expression of plant defense genes and induce plant chemical defenses [52,53]. In this study, *AmGASA1*-*7*, *10*, *12*, *13*, *15*, *18*, *21*, *23*, and *25* had MeJA and defense regulatory elements upstream, consistent with their up-regulated expression after pathogenic microorganism infection. Not all Snakin/GASA family members of the three mangrove species with MeJA and defense regulatory elements were up-regulated after infection by pathogenic microorganisms. Salicylic acid (SA) can act as a signal to promote the expression of downstream defense genes and limit the growth of pathogenic microorganisms [54,55]. Four SA regulatory elements were upstream of *KoGASA4*, and its expression was up-regulated after pathogenic microorganism infection. KoGASA4 is a secreted protein with a signal peptide and was predicted to localize in the extracellular milieu. Therefore, KoGASA4 has some characteristics of plant HDPs, and its function is worthy of further verification. Although *AcGASA6* had two SA upstream regulatory elements and its expression could be induced by pathogenic microorganisms, it did not have a signal peptide and was predicted to localize in the nucleus. Therefore, after pathogenic microorganisms have invaded the plants, the increased level of SA induces the expression of *AcGASA6*. A recent report has shown that the type one protein phosphatases (TOPP)-SnRK2 module of ABA signaling in *A. thaliana* was disturbed by the pathogenic effector AvrE, resulting in up-regulation of ABA signaling and stomatal closure that promoted the generation of interstitial waterlogging and pathogenic infection [56]. Four ABA regulatory elements upstream of *AcGASA10* may account for its up-regulation after infection by pathogenic microorganisms. The regulatory relationship between the Snakin/GASA family members of the three mangrove species and hormones should be further investigated.

Based on prediction, the structure of Snakin/GASA family members is highly conserved [39]. The structures of these Snakin/GASA family members were similar to previous studies [39,42]. These results confirmed the structural conservation of Snakin/GASA family members. Furthermore, the 3D structures of Snakin/GASA family members in the three mangrove species were similar to those of potato Snakins [13].

Microorganisms often cause diseases in plants. For instance, *T. texanus* can cause browning of the leaf edges and veins, *Jattaea* spp. will cause yellowing and ulceration of plant leaves; and *Fusarium* spp. will cause the plant to wilt [57,58,59]. Genes involved in plant pathogen defense are often up-regulated after microbial infection. The synthesis of most HDPs occurs precisely under conditions of infection or other stress factors. Multiple studies have shown that Snakin/GASA family members are involved in pathogen defense. For example, overexpression of the Snakin-1 gene enhances resistance to *Rhizoctonia solani* and *Erwinia carotovora* in transgenic potato plants [60]. Expression of Snakin-2 is up-regulated after infection of potato tubers with the compatible fungus *B. cinerea* [61]. The new *CaSn* gene from the Snakin family induces resistance against root-knot nematode infection in *Capsicum annuum* [62]. Snakin-3 was induced 24 h after infection by *Pseudomonas syringae* pv. *Tabaci* [13]. *PnSN1* in *P. notoginseng* roots was induced 24 h after infection by *F. solani* [17]. *TcGASA12* and *TcGASA13* of *Theobroma cacao* were up-regulated after infection by *Phytophthora megakarya* [42]. Snakin/GASA family members whose expression was not induced after being infected by pathogenic microorganisms did not necessarily lack antibacterial activity. The Snakin/GASA family members whose expression is induced by pathogens are more likely to have functions involved in plant defense and antibacterial effects. According to the RT-qPCR results of the infected leaves, the expression levels of *KoGASA3*, *4*, *AcGASA5*, *6*, *9*, *10*, *AmGASA1*-*5*, *7*, *12*, *13*, *15*, *18*, *23*, and *25* were significantly higher than those of normal leaves. They may be candidates for HDP and deserve further verification. The prediction of HDPs relies on multiple criteria, including sequence features such as the presence of conserved motifs, charge, hydrophobicity, and secondary structure. Additionally, subcellular localization and phylogenetic analysis provide valuable information on the potential function of the protein. Based on these criteria, it is suggested that AcGASA6 may not be an HDP, as it lacks a signal peptide and is predicted to be localized in the nucleus. Regarding AcGASA9, its GASA domain is incomplete, which may affect its functionality as an HDP. AmGASA12 was predicted to be located in chloroplasts, and its GASA domain is incomplete. According to sequence characteristics, amino acid number, and subcellular localization prediction, they may not be HDPs. *AmGASA1-5*, *7*, *13*, *15*, *18*, *23*, and *25* can be regulated by ABA or MeJA, while *KoGASA3*, *4*, and *AcGASA5*, *10* are regulated by ABA. These genes may be induced by pathogenic microorganisms, resulting in increased expression. Thus, they may play a role in the plant’s defense against pathogenic microorganisms. Further verification is needed to confirm their functions. That is very worthy of study; there has been much discussion and in-depth excavation, and it is also one of the directions for future work.

## 5. Conclusions

In this study, the number of Snakin/GASA family members varied among the three mangrove species. Specifically, *K. obovata*, *A. corniculatum*, and *A. marina* had 9, 13, and 27 candidate Snakin/GASA family members, respectively. Sequence alignment showed that the cysteine residues in the GASA domains of Snakin/GASA family members were relatively conserved within the three mangrove species. Additionally, the peptide lengths of the Snakin/GASA family members in the three mangrove species were different. Through evolutionary analysis, prediction of physicochemical properties, and qRT-PCR results, we have identified several potential candidates for HDPs. Taken together, these findings suggest that *AmGASA1*-*5*, *7*, *13*, *15*, *18*, *23*, *25*, *KoGASA3*, *4*, *AcGASA5*, and *10* are most likely involved in plant defense. These candidates warrant further investigation to determine their involvement in plant defenses. This study lays the foundation for further investigation and exploration of the HDPs present in mangrove plants.

## Figures and Tables

**Table 1:** Physicochemical properties prediction of Snakin/GASA family members in *A. corniculatum*, *K. obovata* and *A. marina*.

Name	Size (aa)	pI	Cys Number	Arg + Lys Number	Instability Index	Aliphatic Index	GRAVY ^1^	SignalP ^2^
AcGASA1	346	8.52	21	46	31.01	74.65	−0.215	No
AcGASA2	105	7.94	13	12	53.3	66	−0.07	Yes
AcGASA3	149	8.52	10	16	35.18	70	−0.295	No
AcGASA4	195	9.59	14	37	47.51	85.54	−0.401	No
AcGASA5	89	8.39	14	13	35.19	58.09	−0.215	Yes
AcGASA6	421	5.86	20	44	43.13	76.53	−0.308	No
AcGASA7	346	8.52	21	46	31.01	74.65	−0.215	No
AcGASA8	387	8.28	22	47	36.84	67.03	−0.357	No
AcGASA9	41	9.2	8	7	39.43	28.54	−0.459	Yes
AcGASA10	97	8.86	12	13	24.48	63.4	−0.005	Yes
AcGASA11	154	9.54	12	25	47.83	65.19	−0.301	Yes
AcGASA12	88	9.14	12	15	37.79	74.32	−0.074	Yes
AcGASA13	71	9.06	12	13	25.8	30.14	−0.586	No
KoGASA1	88	9.22	13	14	27.06	58.86	−0.115	Yes
KoGASA2	156	9.21	15	17	60.48	78.21	0.085	No
KoGASA3	187	9.68	13	27	62.17	62.09	−0.34	Yes
KoGASA4	94	9.08	14	13	29.43	67.45	−0.014	Yes
KoGASA5	96	8.76	12	12	44.85	45.83	−0.248	Yes
KoGASA6	107	9.42	12	15	49.69	47.38	−0.308	Yes
KoGASA7	100	8.79	12	12	49.54	68.4	−0.082	Yes
KoGASA8	88	8.46	12	12	25.46	70.8	0.043	Yes
KoGASA9	186	8.22	13	22	33.36	58.23	−0.533	No
AmGASA1	111	9.33	14	17	37.8	50.99	−0.214	Yes
AmGASA2	96	8.92	13	13	45.97	67.19	0.006	Yes
AmGASA3	116	8.53	11	11	69.38	59.83	−0.097	No
AmGASA4	159	9.83	13	25	87.11	55.16	−0.457	Yes
AmGASA5	88	9.02	12	14	27.62	65.34	−0.051	Yes
AmGASA6	114	9.24	13	14	62.34	53.07	−0.235	Yes
AmGASA7	104	9.34	12	15	60.22	64.9	−0.154	Yes
AmGASA8	103	8.79	12	12	38.72	52.47	−0.378	No
AmGASA9	105	9.06	12	15	40.01	78.86	−0.030	Yes
AmGASA10	107	8.45	13	12	33.71	77.48	0.121	Yes
AmGASA11	98	9.28	12	15	36.42	54.8	−0.207	Yes
AmGASA12	249	9.72	10	23	78.37	75.98	−0.086	Yes
AmGASA13	106	9.52	12	16	39.7	47.83	−0.284	Yes
AmGASA14	111	9.67	12	18	34.84	54.59	−0.307	Yes
AmGASA15	113	8.17	15	12	80.39	60.44	−0.251	Yes
AmGASA16	89	9.62	12	11	32.96	70.11	−0.085	Yes
AmGASA17	88	8.74	12	13	30.24	63.18	−0.23	Yes
AmGASA18	110	9.35	12	15	42.5	66.55	−0.03	Yes
AmGASA19	307	5.39	10	44	30.99	64.85	−0.523	Yes
AmGASA20	108	9.67	12	19	38.1	76.67	0.024	Yes
AmGASA21	89	8.88	12	15	45.91	59.21	−0.307	Yes
AmGASA22	87	8.76	12	13	36.24	66.21	−0.093	Yes
AmGASA23	96	9.15	13	14	44.89	63.02	0.050	Yes
AmGASA24	156	9.34	13	20	74.42	52.5	−0.374	Yes
AmGASA25	173	7.08	10	18	41.44	66.65	−0.125	Yes
AmGASA26	100	9.18	12	14	36.35	74.2	0.065	Yes
AmGASA27	109	8.96	11	13	44.47	80.55	−0.049	Yes

^1^ GRAVY: grand average of hydropathicity. ^2^ SignalP5.0: prediction, yes: have signal peptide, no: no signal peptide.

**Table 2:** Gene duplication events in *A. corniculatum* and *K. obovata*.

Duplicated Gene Pairs	*Ka*	*Ks*	*Ka/Ks*	Duplicated Type	Time (MYE)
*AcGASA7*&*AcGASA1*	0.89	0.80	1.11	Tandem	29.67
*AcGASA8*&*AcGASA5*	1.06	0.82	1.30	Tandem	35.37
*AcGASA8*&*AcGASA4*	1.01	0.96	1.06	Tandem	33.74
*AcGASA5*&*AcGASA6*	1.00	1.00	1.00	Tandem	33.36
*AcGASA5*&*AcGASA3*	1.05	0.86	1.22	Tandem	34.92
*AcGASA5*&*AcGASA9*	0.99	1.02	0.97	Tandem	33.13
*AcGASA6*&*AcGASA2*	1.03	0.87	1.18	Tandem	34.48
*AcGASA6*&*AcGASA4*	1.01	0.96	1.05	Tandem	33.65
*AcGASA2*&*AcGASA3*	1.05	0.85	1.24	Tandem	35.10
*AcGASA4*&*AcGASA3*	0.97	1.09	0.89	Tandem	32.38
*KoGASA1*&*KoGASA5*	0.74	1.82	0.61	Segmental	60.75
*KoGASA1*&*KoGASA8*	1.02	0.96	0.32	Segmental	31.89

**Table 3:** Synteny analysis of Snakin/GASA family members between *K. obovata*, *A. corniculatum*, *V. vinifera*, *P. trichocarpa*, and *A. thaliana*.

Relationship	Members	Location ^1^	Members	Location ^1^
*A. thaliana* and	*AtGASA14*	3	AcGASA9	3
*A. corniculatum*	*AtGASA13*	5	*AcGASA11*	10
*A. thaliana* and	*AtGASA1*	1	*KoGASA7*	6
*K. obovata*	*AtGASA7*	2	*KoGASA1*	1
	*AtGASA13*	5	*KoGASA3*	2
	*AtGASA4*	5	*KoGASA6*	4
	*AcGASA11*	10	*KoGASA3*	4
	*AcGASA12*	16	*KoGASA8*	7
	*AcGASA10*	7	*KoGASA5*	4
*A. corniculatum*	*AcGASA11*	10	*VvGASA5*	14
and *V. vinifera*	*AcGASA12*	16	*VvGASA9*	18
	*AcGASA13*	18	*VvGASA9*	18
*A. corniculatum*	*AcGASA13*	18	*PtGASA16*	14
and *P. trichocarpa*	*AcGASA9*	3	*PtGASA10*	6
*K. obovata* and	*KoGASA1*	1	*PtGASA2*	1
*P. trichocarpa*	*KoGASA1*	1	*PtGASA13*	9
	*KoGASA9*	14	*PtGASA17*	15
	*KoGASA3*	2	*PtGASA4*	1
	*KoGASA6*	4	*PtGASA18*	17
	*KoGASA4*	4	*PtGASA19*	17
	*KoGASA7*	6	*PtGASA6*	2
	*KoGASA7*	6	*PtGASA9*	5
	*KoGASA8*	7	*PtGASA16*	14
*K. obovata* and	*KoGASA1*	1	*VvGASA2*	3
*V. vinifera*	*KoGASA9*	14	*VvGASA1*	1
	*KoGASA9*	14	*VvGASA8*	17
	*KoGASA3*	2	*VvGASA5*	14
	*KoGASA6*	4	*VvGASA6*	14
	*KoGASA6*	4	*VvGASA7*	17
	*KoGASA8*	7	*VvGASA9*	18

^1^ Location of members on chromosomes.

## Data Availability

The authors confirm that the data supporting the results of this study can be found within the article or its Supplementary Materials.

## Supplementary Materials

The following supporting information can be downloaded at: https://www.mdpi.com/article/10.3390/genes14040923/s1, Figure S1: Normal leaves and leaves infected with pathogenic microorganisms of *A. corniculatum*, *K. obovata* and *A. marina*; Table S1: Primers used for qRT-PCR; Table S2: Reference genes were used in qRT-PCR; Table S3: Subcellular localization prediction of Snakin/GASA family members in *A. marina*, *K. obovata* and *A. corniculatum*; Table S4: Microbial detection of infected leaves. Table S5: Identification of pathogenic microorganism.
