# Supplementary material for: Genome-Wide Identification and Bioinformatics Analyses of Host Defense Peptides Snakin/GASA in Mangrove Plants
Source: Genes (Basel). 2023 Apr 16;14(4):923. doi: 10.3390/genes14040923 (PMC10137857; doi:10.3390/genes14040923)
Supplement: Supplementary file 1 [file genes-14-00923-s001.zip › genes-2257908-supplementary.pdf]

Supplementary Material

Genome-wide identification and bioinformatics analyses of host defense peptides Snakin/GASA in mangrove plants

Chenjing Shang [1,*], TingYe [1], Qiao Zhou [1], Pengyu Chen [1,2], Xiangyu Li [1,2], Wenyi Li [3], Si Chen [1], Zhangli Hu [1], Wei Zhang [1,*]

***Correspondence:**
cjshang@szu.edu.cn, Tel.: +86 755 26958849 (C.S.);
zhangwei1994@szu.edu.cn, Tel.: +86 755 26958849 (W.Z.)

1. Supplementary Figures and Tables

1.1. Supplementary Figures

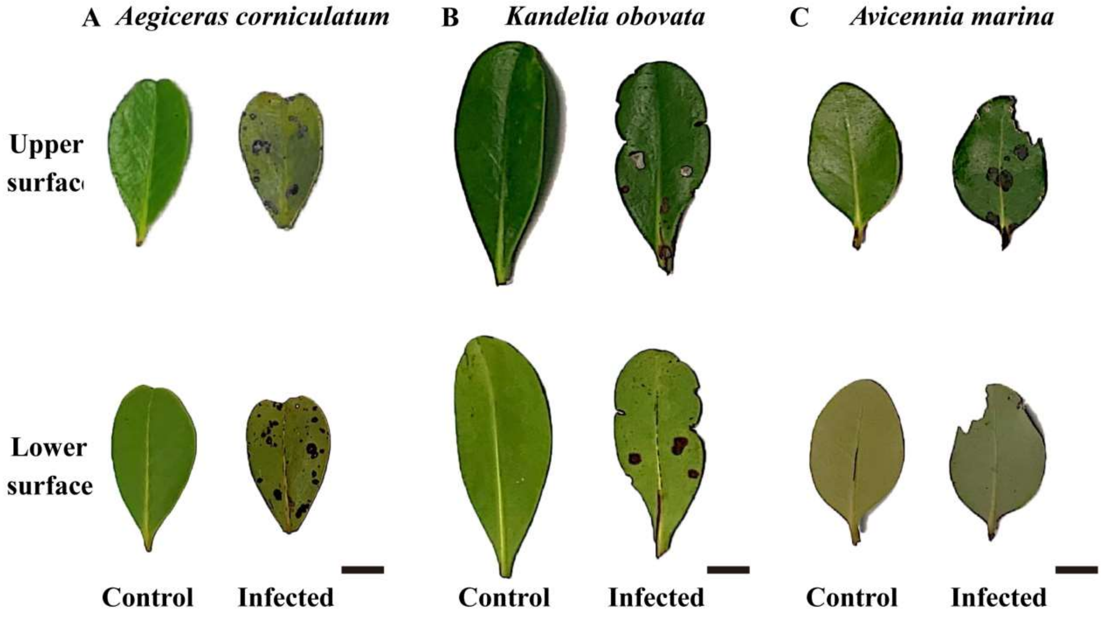

Figure S1: Normal leaves and leaves infected with pathogenic microorganisms of *A. corniculatum*, *K. obovata* and *A. marina*. The scales are 1 cm.

1.2. Supplementary Tables

Table S1: Primers used for qRT-PCR.

Gene	Primer -F (5′-3′)	Primer -R (5′-3′)
KoGASA1	TGGAACCTGCTATGGCTCAAC	AGAGGGAACACACTTGCACT
KoGASA2	AGATGCACCACAAGATGCTC	GCTTGTTTCCGTAGGTTCCAG
KoGASA3	CTTTGTGGGGAGAGGTGCAA	CATAAGTCCCTGGTGGCACA
KoGASA4	AGCTCCATCGCTCAGATTGC	GCATTTGGAGCAGCACTTCA
KoGASA5	CAAGGCAGGTTACCAGGACC	ATAGCACGGGCATTCTGACTT
KoGASA6	CCCAATGCCCATCACAATGC	GCACACGGCCTTATTCCCAT
KoGASA7	TTGTCTTCCAGGCCAAACCTT	AGCAGGGGCAGACATCGTA
KoGASA8	GCGGGGTTAGGTGCTCAAAA	AAGGGCACTCGTCCTTGTTA
KoGASA9	AATGGGATTGTGTTCATCCGGT	CTCATTTGCTTGCTCCCACC
AcGASA1	GAGCTCTTGTTTGTTTGAGCCC	ATTTGAGGCACCGGTCTTGT
AcGASA2	AGATGCTCAAAGGCAGCGAG	TTCGAGGTGCAATTAAACGCTG
AcGASA3	AAGAACCAAGCACCACCTCA	CCCGAGCTGAGCTAAAACCT
AcGASA4	AAAGGAACATGAAGATTCGAGAACG	GTGGTGCTTGGTTCCTGCT
AcGASA5	GCGAGAGATGCTCGGAGG	CAAGGGCACTCGTCCTTGTT
AcGASA6	ATCAACCGAGTCAGTCTTCACAA	CACACTCCTGCCTTTCGTCC
AcGASA7	TGAGCTCTGGTTTGTTTGAGC	TATAAATGCCATTGAGGCACCG
AcGASA8	GAGCTCTGGTTTGTTTGAGCC	ATAAATGCCATTGAGGCACCG
AcGASA9	AAATGTTTGTGTGTGCCACCT	CCTCCTCTTTTGGTCTTCCAGTT
AcGASA10	ATGGCCTATAAGCCCATGCC	ATCCCGCAATATTCCAAGCATC
AcGASA11	CACCACCAACTCCAGTGACC	CAAAGGGGAACGCAATCGAA
AcGASA12	TTCATCTCTCATCCAGGCGAC	GCAGCATATCCCGCAATACTTC
AcGASA13	GGGTAAAAGCCAGGTGCAAGA	CTTCTTGTTCTCATAGCAAGGGC
AmGASA1	GACACAGGCATTCGGGCTT	GCATGGCTTCTTGTATGCTGT
AmGASA2	ATCTTGCAGACGCTATGCCT	CAGCAGTACTTGACGCAACG
AmGASA3	CCTGTCCATGCTCCAAAGGG	TAAATGCACACATTGCCCCAC
AmGASA4	CCTTCTCCACCAGTTAAGGCAT	CTCACGGCGCATAGTGGAG
AmGASA5	CTTCACTGGTTCACACCACAC	ACATTCCTCACAGCAGATTCCA
AmGASA6	AGCCTTCAACCTCAGGAGTG	TTGTTCCCATACGTGCCTGG
AmGASA7	GCCACGTCAGAAACTGCATAC	CTGCACAGATTTGGCCTTCTT
AmGASA10	CAGCAACGATGGTGAAAGGTG	TTGCCTCGAGTGTAAGCTGC
AmGASA12	CAGCATCAAGTACCACCACA	ACACATTTGGCCTTGGCAC
AmGASA13	ATACCACAAGCCCTGCATGTT	AGTTGTTGTAGCAAGGGCAAAC
AmGASA15	CGAAGACCGTTTACCTCCTCA	CTTTGAGCATCGGCGCTTG
AmGASA18	TCGAAGGCCACGATCAAGAG	TCTTCTGTCTCGACGCCAAT
AmGASA21	CTCAAAGTGCGCTGTTCGG	AAGGGCACTCGTCCTTGTG
AmGASA23	GCTGTCCCTGTCCCAACTC	AAGCAACGATCCCACCTTCC
AmGASA25	CATCATGGCAAACCGCCAG	GCGTAGGCCGGAGTCTCT

Table S2: Reference genes were used in qRT-PCR.

Internal reference	Primer -F (5′-3′)	Primer -R (5′-3′)
β-Actin of *A. corniculatum*	AGCTCATCGGTGGAGAAGAA	GTTGGAACAGGACCTCAGGA
18SrRNA of *A. corniculatum*	ACCATAAACGATGCCGACCAG	TTCAGCCTTGCGACCATACTC
β-Actin of *A. marina*	AGATGGCAGAAGCTGAGGA	CCATGCCAACCATCACACC
18SrRNA of *A. marina*	ACCATAAACGATGCCGACCAG	CCTTTAAGTTTCAGCCTTGCG
GAPDH of *K. obovata*	TCGTCCAGGTCTCCAAG	AGCCAGATCCACCACTC
18SrRNA of *K. obovata*	CCTGAGAAACGGCTACCACATC	ACCCATCCCAAGGTCCAACTAC

Table S3: Subcellular localization prediction of Snakin/GASA family members in *A. marina, K. obovata* and *A. corniculatum*.

Proteins	WoLF PSORT	Plant-PLoc
AcGASA1	Chloroplast	Extracellular
AcGASA2	Extracellular	Extracellular
AcGASA3	Chloroplast	Chloroplast
AcGASA4	Chloroplast	Chloroplast
AcGASA5	Extracellular	Extracellular
AcGASA6	Nucleus	Nucleus
AcGASA7	Cytoskeleton	Chloroplast
AcGASA8	Chloroplast	Chloroplast
AcGASA9	Chloroplast	None
AcGASA10	Extracellular	Extracellular
AcGASA11	Extracellular	Chloroplast
AcGASA12	Extracellular	Extracellular
AcGASA13	Chloroplast	Extracellular
KoGASA1	Extracellular	Golgi apparatus
KoGASA2	Extracellular	Extracellular
KoGASA3	Extracellular	Chloroplast
KoGASA4	Extracellular	Extracellular
KoGASA5	Extracellular	Extracellular
KoGASA6	Extracellular	Extracellular
KoGASA7	Extracellular	Extracellular
KoGASA8	Extracellular	Extracellular
KoGASA9	Mitochondrion	Chloroplast
AmGASA1	Extracellular	Extracellular
AmGASA2	Chloroplast	Extracellular
AmGASA3	Chloroplast	Cytoplasm
AmGASA4	Extracellular	Chloroplast

AmGASA5	Extracellular	Extracellular
AmGASA6	Extracellular	Extracellular
AmGASA7	Mitochondrion	Extracellular
AmGASA8	Extracellular	Extracellular
AmGASA9	Extracellular	Extracellular
AmGASA10	Extracellular	Extracellular
AmGASA11	Extracellular	Extracellular
AmGASA12	Chloroplast	Chloroplast
AmGASA13	Chloroplast	Extracellular
AmGASA14	Extracellular	Extracellular
AmGASA15	Extracellular	Extracellular
AmGASA16	Extracellular	Extracellular
AmGASA17	Extracellular	Extracellular
AmGASA18	Extracellular	Extracellular
AmGASA19	Extracellular	Chloroplast
AmGASA20	Extracellular	Extracellular
AmGASA21	Extracellular	Extracellular
AmGASA22	Chloroplast	Extracellular
AmGASA23	Extracellular	Extracellular
AmGASA24	Extracellular	Chloroplast
AmGASA25	Extracellular	Chloroplast
AmGASA 26	Chloroplast	Extracellular
AmGASA 27	Extracellular	Extracellular

Table S4: Microbial detection of infected leaves.

Sample species	Infected fungal species	Infection symptoms
A. corniculatum	*Tropicoporus texanus*	marginal and/or interveinal scorching [1]
K. obovata	*Jattaea* spp.	yellow and/or ulcerated [2]
A. marina	*Berkeleyomyces basicola*	Wilt and/or black root rot [3]

Table S5: Identification of pathogenic microorganism

Species	Sequencing results of microbial PCR assays on infected leaves	NCBI search result Scientific Name	E value	Per. ident
A. corniculatum	CGCTCGATTCAGTTGGCTCTTTGACTTTACACACCTGTGCACCTTCGGTCTGAATTTTCTTGCTTGAATTTTTATTCAAGTC	*Dichostereum eburneum*	3.00E-65	94.90%
	CGCTCCCTGGAGTGGGATTAGAGTGTTGCGTTGGATTTATTTCCATGGCTCACTCTGCAAGAAAATCGTCCGTTTTTTTAC			
	ACCTATTTTGTATGTCTTTGAATTTAATCGATTGCTCCGTGATGGGGTGATTGTAAAAATAAATACAACTTTCAGCAACGG			
	ATCTCTTGGCTCTCCCATCGATGAAGAACGCAGCGAACTGCGATACGTAATGTGAATTGCAGAATTCAGTGAATCATCG			
	AATCTTTGAACGCATCTTGCGCCGCTCGGTTTTCCGAGGGGCACGTCTGTTTGAGTGTCACGTTAATCTATCACCCACCTT			
	TTGGTGAAGTGGACTTGGACTGTGCTGTCATTTATTTGACGGCTCGTCTCGAAATGCATTGTTGCCTGCAGAGTCTTGCGC			
	GGAGCGAGACTGATGAAACGGTGTGATATTTATATCTGCGCTGAAGTAGTCGGTTTTGAGCTGCTGCACGAGTTGCACTC			
	TGGGCTTATAAAATCGAGGATTTTAATTAATCCCCAATTTCTTTGCGTTTGACCTCAAATCAGATGGGACT			
K. obovata	CCGGGGGCCGCGGCGTGGGCGAGTCCTCTCGCCGTCGGCCCCATCCCCGTCCGACGCGCTCCCGTGCGTGTCGTGGCGG	*Jattaea discreta*	3.00E-29	87.27%
	CCAACGAACCCCGGCGCGGAACGCGCCAAGGAAAACCGAAACGAAGCGTCAGCCTCCCGCCGGCCCCGTTCGCGGTG	*Jattaea leucospermi*	3.00E-29	87.27%
	CGCTCGGGTGGGGGGACGAGGCGTCTCTTGAATGTCAAAACGACTCTCGGCAACGGATATCTCGGCTCTCGCATCGATG	*Jattaea tumidula*	3.00E-29	87.27%
	AAGAACGTAGCGAAATGCGATACTTGGTGTGAATTGCAGAATCCCGTGAACCATCGAGTCTTTGAACGCAAGTTGCGCC	*Jattaea taediosa*	3.00E-29	87.27%
	CGAAGCCATCAGGCCAGGGCACGTCTGCCTGGGCGTCACGCATAGCGTCGCCCCCTCTCCCTCTCCCACGGAGCGGAAC	*Jattaea ribicola*	3.00E-29	87.27%
	CGGAGGGGGCGGACATTGGCCTCCCGTGCTCCCTCGGGCGCGGCCGGCCCAAATGCGATCCCTCGGCGACGCACGTCG	*Jattaea mookgoponga*	3.00E-29	87.27%
	CGACCAGTGGTGGTTGAAAACTCAACTCGCGTGCTGGCTGTCGTGTTCAACGACGTCGTCCGATCGGGCATCGAGAGCG	*Jattaea aphanospora*	3.00E-29	87.27%
	ACCCAACGGCGCGATGCGCCTTCGACCGCGACCCCAGGTCAGGCGGGATCAC			
A. marina	CCACCCGGGGGCCGCGGCGTGGGCGAGTCCTCTCGCCGTCGGCCCCATCCCCGTCCGACGCGCTCCCGTGCGTGTCGTG	*Berkeleyomyces basicola*	2.00E-36	88.14%
	GCGGCCAACGAACCCCGGCGCGGAACGCGCCAAGGAAAACCGAAACGAAGCGTCAGCCTCCCGCCGGCCCCGTTCGC			
	GGTGCGCTCGGGTGGGGGGACGAGGCGTCTCTTGAATGTCAAAACGACTCTCGGCAACGGATATCTCGGCTCTCGCATC			
	GATGAAGAACGTAGCGAAATGCGATACTTGGTGTGAATTGCAGAATCCCGTGAACCATCGAGTCTTTGAACGCAAGTTG			
	CGCCCGAAGCCATCAGGCCAGGGCACGTCTGCCTGGGCGTCACGCATAGCGTCGCCCCCTCTCCCTCTCCCACGGAGCG			

GAACCGGAGGGGGCGGACATTGGCCTCCCGTGCTCCCTCGGGCGCGGCCGGCCCAAATGCGATCCCTCGGCGACGCAC

GTCGCGACCAGTGGTGGTTGAAAACTCAACTCGCGTGCTGGCTGTCGTGTTCAACGACGTCGTCCGATCGGGCATCGAG

AGCGACCCAACGGCGCGATGCGCCTTCGACCGCGACCCCAGGTCAGGCGGGAT

References

1. Brown, A.A.; Lawrence, D.P.; Baumgartner, K. Role of Basidiomycete Fungi in the Grapevine Trunk Disease Esca. *Plant Pathol* **2020**, *69*, 205–220.
2. Kazemzadeh-Chakusary, M.; Mohammadi, H.; Sohrabi, M. Occurrence and Pathogenicity of *Jattaea Algeriensis* (*Calosphaeriales*, *Calosphaeriaceae*) and *Biscogniauxia Mediterranea* on Forest Trees in Guilan Province (Iran). *Journal of Phytopathology* **2021**, *169*, 129–138.
3. Li, X.; He, S.; Gao, Y.; Xing, S.; Ren, H.; Yang, H.; Gao, Y.; Wang, J.; Li, N.; Duan, J. Ceratocystis and Related Genera Causing Wilt of *Cunninghamia Lanceolata* Yunnan, China. *For Pathol* **2022**, *52*, e12744.
